# Response of Spatial Patterns of Denitrifying Bacteria Communities to Water Properties in the Stream Inlets at Dianchi Lake, China

**DOI:** 10.1155/2015/572121

**Published:** 2015-10-04

**Authors:** Neng Yi, Yan Gao, Zhenhua Zhang, Yan Wang, Xinhong Liu, Li Zhang, Shaohua Yan

**Affiliations:** ^1^Institute of Agricultural Resources and Environment, Jiangsu Academy of Agricultural Sciences, 50 Zhongling Street, Nanjing 210014, China; ^2^Key Lab of Food Quality and Safety of Jiangsu Province, State Key Laboratory Breeding Base, 50 Zhongling Street, Nanjing 210014, China

## Abstract

Streams are an important sink for anthropogenic N owing to their hydrological connections with terrestrial systems, but main factors influencing the community structure and abundance of denitrifiers in stream water remain unclear. To elucidate the potential impact of varying water properties of different streams on denitrifiers, the abundance and community of three denitrifying genes coding for nitrite (*nirK, nirS*) and nitrous oxide (*nosZ*) reductase were investigated in 11 streams inlets at the north part of Dianchi Lake. The DGGE results showed the significant pairwise differences in community structure of *nirK, nirS*, and *nosZ* genes among different streams. The results of redundancy analysis (RDA) confirmed that nitrogen and phosphorus concentrations, pH, and temperature in waters were the main environmental factors leading to a significant alteration in the community structure of denitrifiers among different streams. The denitrifying community size was assessed by quantitative PCR (*q*PCR) of the *nirS, nirK*, and *nosZ* genes. The abundance of *nirK, nirS*, and *nosZ* was positively associated with concentrations of total N (TN) and PO_4_^3−^ (*p* < 0.001). The difference in spatial patterns between *nirK* and *nirS* community diversity, in combination with the spatial distribution of the *nirS/nirK* ratio, indicated the occurrence of habitat selection for these two types of denitrifiers in the different streams. The results indicated that the varying of N species and PO_4_^3−^ together with pH and temperature would be the main factors shaping the community structure of denitrifiers. Meanwhile, the levels of N in water, together with PO_4_^3−^, tend to affect the abundance of denitrifiers.

## 1. Introduction

It has been commonly observed that a large but variable proportion of aquatic N removal occurred in freshwater ecosystems, including groundwater, streams, lakes, and wetlands [1, 2]. The variation in microbial abundance and community structure among different aquatic ecosystems has been recognized as one of the most important factors which contributed to the changing of N biogeochemical cycling process in different aquatic ecosystems [3, 4]. Therefore, studying the spatial patterns of functional microbial guilds can help us to understand the relationships between microbial community ecology and the related ecosystem functions. Canonical denitrification has been generally considered as the main mechanism for permanent removal of N from aquatic ecosystem through returning of N in water to the atmosphere in the form of N_2_O and N_2_, although some alternative pathway, such as anaerobic ammonium oxidation (Anammox), has been discovered [5]. Denitrification in aquatic ecosystems has been widely found removing large proportion of the total N inputs to watersheds and thus providing a valuable ecosystem service by alleviating the impact of increased human N inputs [1]. Several studies have reported that the function of denitrifying bacteria communities was correlated with their abundance and community structure [6–8]. Hence, studying the variation in abundance and structure of denitrifying community will help to understand the variable denitrification potential as well as variable proportion of aquatic N removal that occurred among different aquatic ecosystems.

Streams are an important sink for anthropogenic N owing to their hydrological connections with terrestrial systems. The microbial communities in streams adapted to changes in the concentration and makeup of organic matter [9] and nutrients [10, 11]. Some recent studies have been concentrated on the link between the freshwater bacterioplankton dynamics and the environmental changes [12]. Numerous studies have reported that denitrifying bacteria can be affected by physical and chemical parameters such as pH, temperature (*T*), dissolved oxygen (DO), and N forms [12, 13] in series of laboratory incubation experiments. However, it is hard to identify the factors driving the variation in abundance and community structure of denitrifying bacteria in complicated aquatic ecosystems. Nowadays, functional markers include nitrite reductase (*nirK* and* nirS*) and nitrous oxide reductase (*nosZ*) genes have been frequently used to analyze the diversity and abundance of denitrifying bacteria community in the processes of denitrification and their response to the changing of environmental factors [8, 14–18]. Based on the analysis of these functional markers, recent research has demonstrated that the variation in the assemblage of* nirS*,* nirK*, and* nosZ* populations in soil was closely related to temperature, pH, and DO [19–21]. Furthermore, the abundance of these denitrifies varied in response to different nitrogen concentrations in soil, and a differential response of denitrifies communities structure to environmental gradients has also been reported [22]. These mean that functional genes of* nirS*,* nirK*, and* nosZ* could be sensitive indicators when studying the response of denitrifier community to variation of environmental gradients in complicated ecosystems. So far, limited studies incorporated the phosphorus concentration into the analysis of environmental gradient resulting in denitrifies community change in freshwater ecosystems [17], despite phosphorus being a vital element influencing microbial spatial patterns [23]. Therefore, in this study, we will focus on the environmental factors that have been reported to be closely related with denitrification and the variation of denitrifier community (e.g., nitrogen forms, nitrogen concentration, pH, water temperature, and DO) and the less addressed factor such as phosphorus concentration.

Dianchi Lake is the sixth largest freshwater lake in China. There are 35 streams radially flowing into the Dianchi Lake, which is a shallow plateau freshwater lake in the south-west of Kunming city, Yunnan province of China. Streams around the lake serve as ecohydrological channels that impose anthropogenic stress on the lake ecosystem and eventually cause water quality deterioration [24]. Due to the sedimentation, land reclamation, and excessive pollution, the water quality of 35 streams continued degrading from the level of drinking water quality in 1975 to the level of landscape-use only water in 2009 [24, 25]. The water quality in the north part of Dianchi Lake is the worst grade of national water quality standard. There were relatively higher NH_4_^+^ and NO_3_^−^ concentration even up to 12–20 mg/L in some rivers. The pH values of all sites were alkalescent [24, 26]. More than 6 sewage treatment plants (STPs) had been in operation near 11 streams in the north lake side in recent years. The effluents from the STPs are a major cause of degraded water quality in the down streams within the basin. Generally, effluents are characterized by high concentrations of nitrogen and organic matter [27]. Along with high concentration of nitrogen, many microorganisms especially denitrifying bacterial community entrained in effluents were domesticated [28].

In the present study, in order to address the response of spatial patterns of denitrifying communities to variation in environmental factors among different streams, we investigated the water properties and the abundance and diversity of denitrifying bacterial community in 11 stream inlets with different pollution sources, some of which were receiving effluents from different sewage treatment plants in the north part of Dianchi Lake, an eutrophic lake located in Southwest China (Table 1). We hypothesized that the variation in pollution sources and effluents types, such as the main eutrophication elements of N and P, may modulate the communities of denitrifiers, which may further lead to changes in the biogeochemical cycling of N in the streams and lake. The results would also provide valuable information on how the abundance and diversities of denitrifiers in various streams responded to the variation of water properties such as nitrogen forms, nitrogen concentration, phosphorus concentration, pH, water temperature, and DO. It was also expected to shed some lights on understanding the variable denitrification potential as well as variable proportion of aquatic N removal that occurred among different streams.

## 2. Materials and Methods

### 2.1. Site Description, Sampling, and Water Properties

**Figure 1:**
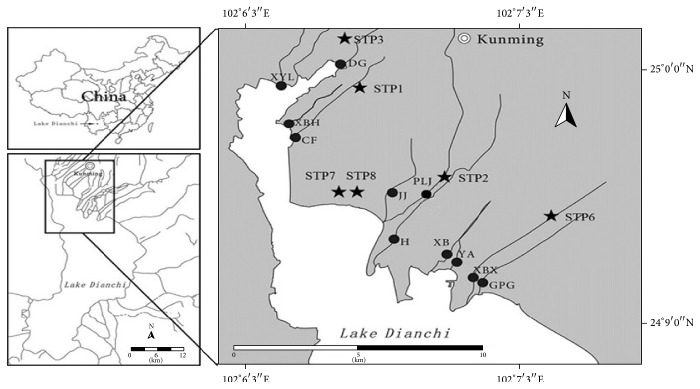
Sampling sites in stream inlets of Dianchi Lake. Black dots (●) are sampling sites; pentagrams (★) are sewage treatment plants (STPs). Stream names: XBX = Xinbaoxiang, H = Haihe, XB = Xiaba, YA = Yaoan, JJ = Jinjia, GPG = Guangpugou, PLJ = Panlongjiang, XBH = Xibahe, CF = Chuangfang, DG = Daguang, and XYL = Xinyunling.

There are 11 streams around Dianchi Lake located from 24°9′ to 25°0′ latitude and 102°6′ to 102°7′ longitude that were investigated (Figure 1).

Dissolved oxygen (DO), oxidation reduction potential (ORP), pH, and water temperature were measured in situ using portable meter (YSI ProPlus, USA) at all sampling sites. Three replicates of surface water (0–0.5 m) samples were randomly collected at three sampling locations from each sampling site of eleven streams using a cylinder sampler on 25 September 2012. Basically, the three sampling locations at each sampling site were from the upper, middle, and lower sections of a stream. One-liter water samples were reserved at −4°C with addition of Hgcl2/acid solution for chemical analysis, and two-liter water samples were filtered immediately for further molecular DNA extraction after being transported to laboratory. The concentrations of total nitrogen (TN), total phosphorus (TP), nitrate (NO_3_^−^), and phosphates (PO_4_^3−^) in the water samples were analyzed using a SEAL AutoAnalyzer 3 (SEAL Analytical Co., Hampshire, UK).

### 2.2. DNA Extraction

All water samples were kept in an ice box, transferred to the laboratory, and filtered through a 5 *μ*m pore size sterilized filter to remove the impurities. The resultant filtrate of each sample (500 mL) was filtered through 0.22 *μ*m Millipore membrane filters using a vacuum air pump and the membranes were stored at −80°C for DNA extraction. The membranes were cut into pieces with sterilized scissors and used immediately for DNA extraction. DNA extractions were performed using an E.Z.N.A. Water DNA Kit (OMEGA Bio-Tek Inc., Doraville, GA, USA) by following the manufacturer's instructions. The DNA samples were stored in a −20°C freezer until use.

### 2.3. Real-Time Polymerase Chain Reaction Assay

The plasmids containing* nirK*,* nirS*, and* nosZ* fragments from environmental samples were used to create standard curve. The PCR amplified products were cloned into vector pMD19-T using the pMD19-T vector system I kit according to the manufacturer's instructions (Takara, Dalian, China). The recombinant plasmids were inoculated into LB broth with ampicillin and incubated at 37°C overnight. Plasmid DNA was then extracted from the correct insert clones of each target gene using the E.Z.N.A. Plasmid Mini Kit II (OMEGA Bio-Tek Inc., Doraville, GA, USA) according to the manufacturer's instructions. The plasmids DNA concentration was determined by NanoVue spectrophotometer (GE Healthcare Europe, Munich, Germany), and then the copy numbers of target genes were calculated. Tenfold serial dilutions of a known copy number of the plasmid DNA were subjected to real-time PCR assay in triplicate to generate an external standard curve.

The real-time polymerase chain reaction (qPCR) was performed on ABI 7500 real-time system (Life technologies, USA) to assess gene abundance. Amplification was performed in a 20-*μ*L reaction mixture using SYBR Premix Ex Taq as PCR Kit provided by the suppliers (Takara bio, Dalian, China). The DNA diluted template corresponding to 1–10 ng of total DNA extracts was used in each reaction mixture. The primers and procedures used to amplify each target gene when performing real-time PCR were listed in Table 2. Data was analyzed using the 7500 software (version 2.0.6, Life technologies, USA). The parameter Ct (threshold cycle) was determined as the cycle number at which a statistically significant increase in the reporter fluorescence was detected. Standard curves for real-time PCR assays were created according to the method described by Henry et al. [34].

### 2.4. PCR Amplification Denaturing Gradient Gel Electrophoresis Analysis

The community structures of* nirK*,* nirS,* and* nosZ* genes were analyzed by denaturing gradient gel electrophoresis (DGGE). The amplification was performed in 50-*μ*L reaction mixtures including 1x PCR buffer, 400 *μ*mol/L of each dNTP, and 2.5 U hot star Taq DNA polymerase (Takara Bio, Otsu, Shiga, Japan) plus primers (Table 2).

The amplified products were pooled and resolved on DGGE gels using a Dcode system (Bio-Rad Laboratories Inc. Hercules, USA). PCR samples (50 *μ*L) containing approximately equal amounts of PCR amplicons were loaded onto the 1 mm thick 8% (w/v) polyacrylamide (37.5 : 1, acrylamide : bisacrylamide) gels in 1x TAE buffer (40 mM Tris-acetate and 1 mM EDTA) with denaturing gradients of 50–75% for 15 h (*nirS*), 50–70% for 12 h (*nirK*), and 50–70% (*nosZ*) (100% denaturant contains 7 mol/L urea and 40% (v/v) formamide) for 15 h at 100 V and 60°C, respectively. After being stained with silver nitrate according to the protocol [35], polaroid pictures of the DGGE gels were scanned using an EPSON (Perfection V700 Photo) scanner and stored as TIFF files and digitized and then analyzed with the Quantity One software (version 4.5, Bio-Rad, USA).

### 2.5. Data Analysis

Three replicates were used in all parameter analysis. Data were presented as the mean values of triplicates and the maximum difference (mean ± SD) among triplicate results was 5%. One way analysis of variance (ANOVA) was performed to test whether there were any significant differences among the means at the 95% confidence level. Potential relationships between all denitrifying bacteria abundance and environmental data sets were tested by Pearson correlation analysis. All data were analysed using SPSS software.

DGGE banding profiles for* nirS*,* nirK,* and* nosZ* communities were digitized after average background subtraction for entire gels. Band position and intensity date for each sample were exported to an excel spreadsheet prior to further statistical analyses. The relative intensity of a specific band was transformed according to the sum of intensities of all bands in a pattern [36]. Redundancy analysis (RDA) for community ordination was conducted using CANOCO (version 4.5, Centre for Biometry, Wageningen, The Netherlands) for Windows using relative band intensity data obtained from the Quantity One analysis [37, 38]. Among all environmental variables, eight parameters, including water temperature, pH, DO, oxidation reduction potential nitrate, total nitrogen, total phosphorus (TP), and phosphates (PO_4_^3−^), were selected to perform RDA by Monte Carlo reduced model tests with 499 unrestricted permutations to statistically evaluate the significance of the first canonical axis and of all canonical axes together. Statistical significance was kept at *p* < 0.05 for all analyses (Table 4).

## 3. Results

### 3.1. Spatial Patterns of Structure and Size of Denitrifier Communities

All digitized data of the three replicates were used in statistics analysis. The Shannon index (*H*) of* nirK*,* nirS*, and* nosZ* calculated from DGGE gels ranged from 2.08 to 2.64, 1.73 to 2.80, and 1.89 to 2.60, respectively. The significant differences among them were observed statistically (*p* < 0.05). There were relative lower richness and diversity of* nirK*,* nirS*, and* nosZ* in Xinyunliang stream (Table 3), while the highest of the Shannon values and richness of* nirK*,* nirS*, and* nosZ* occurred in Guangpugou stream.

**Figure 2:**
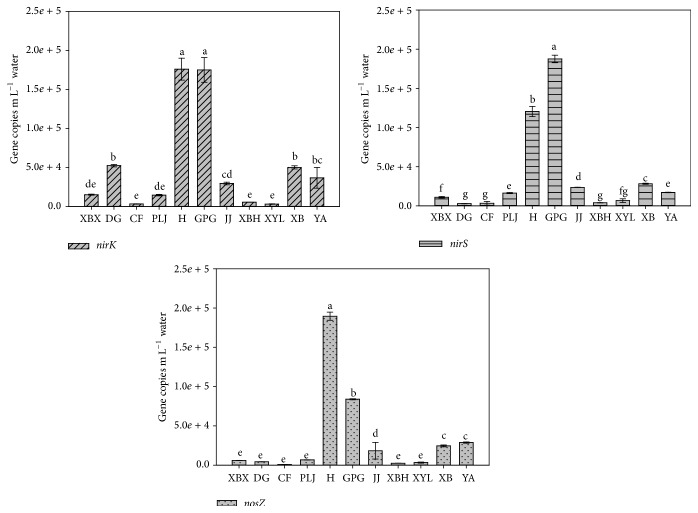
Abundance of* nirS, nirK*, and* nosZ* genes in the water samples. Error bars indicate standard deviations (*n* = 3). The different letters indicate significant differences (*p* < 0.05). Stream names: XBX = Xinbaoxiang, H = Haihe, XB = Xiaba, YA = Yaoan, JJ = Jinjia, GPG = Guangpugou, PLJ = Panlongjiang, XBH = Xibahe, CF = Chuangfang, DG = Daguang, and XYL = Xinyunling.

The results of qPCR showed that the abundance of* nirS, nirK, *and* nosZ* gene copies per mL water ranged from 2.79 × 10^3^ to 1.20 × 10^5^, 3.23 × 10^3^ to 1.76 × 10^5^, and 8.66 × 10^2^ to 1.90 × 10^5^, respectively. The abundance of* nirK, nirS,* and* nosZ* denitrifiers in Xinbaoxiang, Daguan, Chuanfang, and Panlongjiang streams was relatively stable and low. The results of* nirK, nirS,* and* nosZ* genes abundance in group Xiaba and Yaoan streams were consistent. The absolute abundance of denitrification genes in the other five streams varied widely (Figure 2). These results indicated that different pollution source would influence the abundance of denitrifiers in streams. The denitrifiers (*nirK*,* nirS,* and* nosZ*) abundance of the water samples in Haihe stream and Guangpugou stream was significantly higher than that in other stream samples (*p* < 0.05). The abundance of* nirK*- and* nosZ*-type denitrifiers had similar trends in the streams, with higher number in Haihe stream and Guangpugou stream and intermediate levels in Xinbaoxiang, Xiaba, Yaoan, Jinjia, Panlongjiang, and Xiaba streams but lower levels in Chuangfang stream, Daguang stream, and Xinyunliang stream. However, the* nirS*-type abundance in all sites did not totally follow this trend and no significant differences were detected in qPCR data among Haihe, Guangpugou, and Daguang streams.

Ratios of* nosZ* abundance to the abundance of* nirK* +* nirS* for all samples varied widely. The highest* nosZ*/(*nirK *+* nirS*) ratio occurred in Haihe stream (0.64), the lowest* nosZ*/(*nirK *+* nirS*) ratio occurred in Daguang stream (0.08), and the* nosZ*/(*nirK* +* nirS*) ratios in the other streams were similar and ranged from 0.13 to 0.35. Ratios of* nirK* abundance* nirK*/*nirS* to* nirS* abundance in different streams were also different. The highest (*nirK*/*nirS*) ratio occurred in Daguang stream (18.79), the lowest (*nirK*/*nirS*) ratios occurred in Xinyunliang stream (0.48), and the (*nirK*/*nirS*) ratios in the other streams were between 0.90 and 2.15. These results implied that the abundance of* nirS* was not always greater than that of* nirK* in stream inlet water column.

### 3.2. Water Parameters Controlling Denitrifier Communities

In order to determine to what extent the eight environmental properties affected the three types of denitrifying genes on their community compositions, DGGE fingerprints were analyzed by redundancy analysis. The results showed that PO_4_^3−^, pH, and water temperature were the relatively important environmental parameters for denitrifiers (Table 5). For* nirK*-type denitrifier, PO_4_^3−^, TN, and pH explained 50% variations of microbial communities. Variation partitioning analysis showed that PO_4_^3−^, TN, and pH separately explained 21% (*p* = 0.002), 19% (*p* = 0.190), and 10% (*p* = 0.398) of the variation, respectively. The analysis did not reveal significant relationship between* nirS*-type denitrifier communities and any environmental parameters. For* nosZ*-type denitrifier, temperature (17%, *p* = 0.048), NO_3_^−^ (13%, *p* = 0.06), and total P (8%, *p* = 0.278) explained 38% variations of microbial communities.

*nirS* abundance was significantly and positively correlated with* nirK* and* nosZ* abundance.* nirK* abundance was significantly correlated in a positive direction with* nosZ* abundance. These results suggested that all of three denitrifiers can interact with each other. The abundance diversification of* nirK*,* nirS*, and* nosZ* was strongly and positively associated with TN and PO_4_^3−^ (*p* < 0.001). The analysis did not reveal significant relationship between pH and any denitrifying bacteria gene abundance. All relationships between* nirS*,* nirK*, and* nosZ* genes abundance and chemical variables were positively correlated except for DO, ORP, T, and NO_3_^−^, which were negatively correlated with the copy numbers of* nirS*,* nirK*, and* nosZ* genes. The NO_3_^−^ concentration was a key parameter influencing the ratios of* nirK* abundance of* nirS*. A significant correlated correlation existed between ratios of* nosZ* abundance to the abundance of* nirK* +* nirS* and the concentration total nitrogen.

## 4. Discussion

### 4.1. The Variation of Community Pattern of Denitrifying Bacteria according to Pollution Sources and Effluent Types of Different Streams

Nitrogen cycle in aquatic ecosystems is predominantly controlled by bacteria, and their activities determine the fate of nitrogen compounds. Meanwhile, environmental conditions that regulate the activity of bacteria determine where each nitrogen transformation process occurs and the degree of exchange among various nitrogen pools. Thus, chemical information of different nitrogen species alone is not sufficient to predict rates of nitrogen transformation processes in the environment, and information concerning characteristics of nitrogen cycling bacterial community under various environmental conditions is essential for understanding the related nitrogen cycle process. With regard to the denitrification process, previous studies have shown that it was regulated by various environmental factors such as oxygen and nitrogen concentration, quality, temperature, and pH [5, 39]. However, how the denitrifying bacteria communities were correlated with the environmental factors in streams, receiving massive amount of nutrients and pollutants, remains unclear.

In this study, we evaluated differences in the genetic makeup of the communities by comparing DGGE profiles for denitrification genes encoding nitrite and nitrous oxide reductase (*nirK*,* nirS*, and* nosZ*). It has been suggested that DGGE was a powerful tool for identifying and comparing the dominant of these communities [40, 41]. The DGGE results in this study revealed the significant pairwise differences in the community structure of denitrifying bacteria containing the* nirK*,* nirS*, or* nosZ* genes among the water samples collected from different streams. When comparing the diversities of denitrifier communities from all sites, similar trends emerged with low richness and diversity in the Xinyunliang stream. According to previous studies, Xinyunliang streams run through a historical area of old Kunming city, where there are many industries (such as Yunnan smelter), high population density, and poor sewage networks [42]. This serious industrial pollution could reduce bacterial diversity and damage microbial ecological system [43, 44], although the concentration of NO_3_^−^, the substrates for denitrification, in Xinyunliang stream was higher than most of other streams with exception of Guangpugou and Haihe streams. Our studied streams differed substantially in the amount of inorganic nutrients which were potentially available to denitrifiers and other microbial populations during the development of microbial community in the water column. The three large streams around Dianchi Lake, Panlongjiang, Daguan, and Chuanfang streams [42], were the important sites receiving effluents from the STPs, with the characteristics of high nitrate concentrations. Nevertheless, the fast-flowing water and irregular discharge of effluents prevented the stream from developing a stable environment for microbial colonization and propagation. Therefore, these streams also represented relatively low diversities of denitrifying bacteria. It has been reported that the high stream flow and nitrate concentration of streams were the major factors controlling the development of planktonic denitrifier populations [45]. On the contrary, the streams of Haihe and Guangpugou were of narrow and slow-flowing, which leading to the long residence time for nitrogen-containing pollutants and well-established hypoxic (~0.2 mg L^−1^ in DO) environment. Therefore, the abundance of denitrifiers was much higher in Haihe and Guangpugou streams than the other streams, which may enhance denitrification in Haihe and Guangpugou streams [46]. This result was consistent with the previous reports that the most transformation of inorganic nitrogen occurred in narrow streams [47]. Simultaneously, an ecological engineering project using* Eichhornia crassipes* for nutrient removal has been conducted in the 11 streams around Dianchi Lake since June 2011. The roots of* E. crassipes* [48, 49] in streams provided a large specific area for denitrifiers to attach, which would benefit the formation of biofilms and therefore may further change or modify the diversity and abundance of denitrifiers in water [50]. Previous studies suggested that microbial biofilms were highly efficient and successful ecological communities that might also contribute to the influence of the headwater streams on streams, estuaries, and even oceans [51]. Therefore, the slow-moving flows such as Haihe and Guangpugou streams could be considered as living zones of transient storage, where roots and other biofilms bring hydrodynamic retention and biochemical processing into close spatial proximity and influence biogeochemical processes and patterns in streams. All of these results coincided with our hypothesis that the pollution sources and effluent types of different streams would modulate the community composition of denitrifying bacteria to a great extent, although a complex picture of denitrifier community similarity emerged depending on which functional denitrification gene was evaluated.

In this study, we found that the distribution of nir-denitrification genes was much patchier, which was consistent with earlier observations in other streams [52, 53]. Studies of changes in composition and diversity of* nirK* and* nirS* genes communities support the hypothesis of niche differentiation among denitrifying bacteria [54–56]. Meanwhile, this study found the abundance of* nirS* was not consistently greater than that of* nirK* in stream inlet water column, which was different from some of the previously published results [53, 57]. However, in a similar way, some of previous studies also suggested that the spatial distribution of* nirS* and* nirK* genes abundance differs in other types of urban streams, reflecting different habitat preferences [52, 53]. The results of the* nirK*/*nirS* ratios suggested that the* nirK*-type denitrifiers might be more abundant than* nirS*-type denitrifiers in Daguang stream (*nirK*/*nirS* ratio, 18.79), in contrast to Panlongjiang stream (*nirK*/*nirS* ratio, 0.90). However, the concentrations of DO in the two streams were both relatively higher (~2.8 mg L^−1^) than others. This contradicted with previous studies that* nirK* often prevailed in conditionally O_2_-exposed environments [53, 58]. This discrepancy probably was due to other environmental parameters such as nitrogen and phosphorus concentrations [21, 53] which varied significantly in the two streams. Even though the* nirK* and* nirS* are functionally equivalent, denitrifying bacteria harboring either nitrite reductase was likely not under the same community assembly rules [59]. Philippot et al. [60] suggested that the existence of the two types of nitrite reductase (*nir*-gene) was due to differential niche preferences. The different community patterns, together with the spatial distribution of the* nirS*/*nirK* abundance ratio, can suggest habitat selection for the* nirS*- and* nirK*-type denitrifiers [17]. In the present study, the different spatial patterns of* nirK* and* nirS* community diversities, in combination with the spatial distribution of the* nirS*/*nirK* ratio, indicated habitat selection for the two types of denitrifiers. Denitrifying organism includes either* nirS* or* nirK*, but not both of the two-type nitrite reductase genes [61, 62], and experiments have shown the two nitrite reductases to be functionally redundant, as one nir-type gene in denitrifying bacteria can be eliminated and replaced by the other type [63]. This, however, did not necessarily indicate that* nirK*-type denitrifiers contributed more or less in denitrification than* nirK*-type ones. Hence, gene expression analysis is necessary to further investigate which is more important in denitrification in the stream inlet water column around Dianchi Lake.

### 4.2. Relationship between Water Properties and Spatial Patterns of Denitrifying Bacteria

In the present study, the RDA charts of* nirK*,* nirS*, and* nosZ* genes indicated that diversity of denitrifying populations had varying response to environmental factors, and the concentrations of P (PO_4_^3−^ or TP) and N (NO_3_^−^ or TN) were the most important environmental factors causing a significant alteration in the denitrifier community structure among different streams by serving as essential nutrients for microorganisms growth in streams. Meanwhile, abundance of all denitrifiers in this study was, by and large, controlled by the water parameters, especially nutrient (phosphorous and nitrogen) concentration (Table 3). Certainly, microbes need phosphorus for their growth and function. Finlay et al. found increasing phosphorous inputs associated with eutrophication can indirectly promote N losses via enhancing denitrification [64]. Additions of P have been demonstrated to increase N removal in whole-ecosystem experiments in both lakes and streams [65], which provides further support for the role of P as an important control over N cycling and fate in freshwater ecosystems. In addition, different responses of community diversities of* nirS*- and* nirK*-denitrifiers to the changes of phosphorous concentrations agreed with a study suggesting that* nirK*-denitrifiers were most sensitive to alteration of phosphorous concentration [21]. Contrary to previous studies [17, 46], our results implied that variation of phosphorous content in water was positively linked to the abundance of* nirS* and* nirK* genes and resulted in shift of community structure of nir-containing denitrifiers populations. This may further clarify the important function of phosphorous in shaping microorganisms structure in environments [23, 46]. However, the mechanisms concerning how phosphorus would affect growth of denitrifying bacteria in water are still not well understood [21]. Further studies are needed to explain underlying mechanism related to the role of P in regulating the denitrifiers' community, although our results have built some supporting evidence for the related phenomenon. In addition, the water temperature and pH were the main factors driving the changes in the denitrifying bacterial community composition among different streams. The genes* nirS*,* nirK*, and* nosZ* abundance was shown by Pearson correlation coefficient (*r*) to be mightily influenced by water temperature and oxidation reduction potential (ORP). The quality of inlet water in 11 streams differed with water origin and pollution sources [24, 25, 42]. It has been shown that the changes in denitrifying community structures responded to their habitat conditions like temperature and DO gradient and N forms [12, 13, 66, 67]. The pH was known to generally affect denitrifier community diversity and richness [15]. Generally, the effect of temperature on driving biogeochemical processes is either to alter the functioning bacteria without changing the microbial communities or restructuring communities, thus modifying the fundamental physiologies [68]. Previous studies suggested that temperature could directly or indirectly affect the communities' diversity and abundance of denitrifying organisms [16, 20, 69–71].

## 5. Conclusions

The results showed that abundance and diversities of denitrifying genes (*nirK*,* nirS*, and* nosZ*) were variable in the streams of Dianchi Lake. Nutrient concentrations (nitrogen and phosphorous), water temperature, and pH were important environmental factors to alter abundance and community structure of the denitrifiers significantly. The different community patterns, together with the spatial distribution of the* nirS*/*nirK* abundance ratio, suggest habitat selection for the* nirS*- and* nirK*-type denitrifiers.

## Figures and Tables

**Table 1:** Water properties of the streams at Dianchi Lake (mean ± SD).

Streams	NO_3_^−^ (mg L^−1^)	TN (mg L^−1^)	PO_4_^3−^ (mg L^−1^)	TP (mg L^−1^)	ORP (mg L^−1^)	DO (mg L^−1^)	pH	*T* (°C)
XBX	4.79 ± 0.39	7.44 ± 0.19	0.33 ± 0.01	0.39 ± 0.01	18.45 ± 1.63	2.15 ± 0.15	7.83 ± 0.06	19.60 ± 0.00
H	0.16 ± 0.02	23.69 ± 0.79	2.07 ± 0.07	2.10 ± 0.07	−156.80 ± 29.56	0.20 ± 0.10	7.90 ± 0.01	18.80 ± 0.00
XB	0.56 ± 0.14	6.35 ± 1.21	0.39 ± 0.01	0.45 ± 0.03	−46.55 ± 9.83	0.55 ± 0.25	7.78 ± 0.06	17.95 ± 0.05
YA	8.99 ± 0.00	15.08 ± 0.50	0.22 ± 0.01	0.28 ± 0.01	−15.95 ± 4.03	1.05 ± 0.35	7.85 ± 0.07	17.60 ± 0.10
JJ	0.43 ± 0.02	8.14 ± 0.54	0.36 ± 0.01	0.41 ± 0.01	59.55 ± 2.19	1.30 ± 0.10	7.90 ± 0.01	19.50 ± 0.00
GPG	0.08 ± 0.02	23.66 ± 0.16	1.78 ± 0.11	1.86 ± 0.04	−225.75 ± 12.80	0.20 ± 0.10	7.83 ± 0.00	17.90 ± 0.00
PLJ	5.86 ± 0.01	8.08 ± 0.19	0.16 ± 0.01	0.28 ± 0.00	48.05 ± 0.78	2.80 ± 0.00	8.03 ± 0.00	19.70 ± 0.00
XBH	5.92 ± 0.25	8.60 ± 0.19	0.13 ± 0.01	0.19 ± 0.01	37.53 ± 1.34	0.60 ± 0.00	7.77 ± 0.00	21.60 ± 0.00
CF	5.09 ± 0.03	8.63 ± 0.10	0.10 ± 0.01	0.13 ± 0.01	49.50 ± 3.25	3.80 ± 0.10	7.90 ± 0.06	21.75 ± 0.05
DG	12.00 ± 0.67	12.91 ± 0.62	0.06 ± 0.00	0.12 ± 0.02	43.90 ± 0.28	2.70 ± 0.13	7.56 ± 0.04	22.15 ± 0.05
XYL	0.08 ± 0.02	21.40 ± 2.78	0.98 ± 0.09	2.52 ± 0.16	−246.75 ± 17.18	0.45 ± 0.05	7.78 ± 0.02	21.55 ± 0.05

Stream names: XBX = Xinbaoxiang, H = Haihe, XB = Xiaba, YA = Yaoan, JJ = Jinjia, GPG = Guangpugou, PLJ = Panlongjiang, XBH = Xibahe, CF = Chuangfang, DG = Daguang, and XYL = Xinyunliang.

(1) TSN = total soluble nitrogen.

**Table 2:** Primers and thermal profiles used for the qPCR and DGGE.

Target gene	primers	Thermal profile
qPCR	*nosZ*-F [29] *nosZ*1622R [29]	qPCR: 94°C/2 min; 6 cycles of 94°C/30 s, 57°C/30 s (−1°C/cycle), and 72°C/45 s; 30 cycles of 94°C/30 s, 52°C/30 s, and 72°C/45 s.
DGGE	*nosZ*-F [29] *nosZ*1622-GC^*∗*^ [30]	DGGE: 94°C/2 min; 10 cycles of 94°C/30 s, 58°C/30 s (−0.5°C/cycle), and 72°C/60 s; 30 cycles of 94°C/30 s, 53°C/30 s, and 72°C/60 s; 72°C/10 min.

qPCR	Cd3aF [31] R3cd [31]	q-PCR: 94°C/2 min; 6 cycles of 94°C/30 s, 57°C/30 s (−1°C/cycle), and 72°C/45 s; 30 cycles of 94°C/30 s, 52°C/30 s, and 72°C/45 s.
DGGE	Cd3aF [31] R3cd-GC^*∗*^ [32]	DGGE: 94°C/2 min; 10 cycles of 94°C/30 s, 57°C/30 s (−0.5°C/cycle), and 72°C/45 s; 30 cycles of 94°C/30 s, 52°C/30 s, and 72°C/45 s; 72°C/10 min.

qPCR	F1aCu [33] R3Cu [33]	q-PCR: 95°C/3 min; 6 cycles of 95°C/30 s, 63°C/30 s (−1°C/cycle), and 72°C/30 s; 32 cycles of 95°C/30 s, 58°C/30 s, and 72°C/30 s.
DGGE	F1aCu [33] R3Cu-GC^*∗*^ [33]	DGGE: 95°C/3 min; 32 cycles of 95°C/30 s, 58°C/30 s, and 72°C/45 s; 72°C/10 m.

^*∗*^(GGCGGCGCGCCGCCCGCCCCGCCCCCGTCGCCC) was attached to the 5′ end of the primers.

**Table 3:** Shannon index (*H*) and richness (*S*) values of *nirK*, *nirS*, and *nosZ* genes.

Streams	*nirK*		*nirS*		*nosZ*	
	*S*	*H*	*S*	*H*	*S*	*H*
XBX	11.00 ± 1.00cd	2.09 ± 0.12b	16.00 ± 1.00b	2.61 ± 0.36ab	14.00 ± 1.00bc	2.56 ± 0.10a
H	13.33 ± 1.53bc	2.33 ± 0.27ab	14.33 ± 0.58b	2.47 ± 0.29ab	11.00 ± 1.00def	1.95 ± 0.19a
XB	11.33 ± 0.58cd	2.20 ± 0.15ab	18.00 ± 1.00a	2.50 ± 0.20a	12.67 ± 0.58cde	2.34 ± 0.22a
YA	11.00 ± 0.00cd	2.10 ± 0.06b	14.33 ± 0.58b	2.42 ± 0.25ab	15.33 ± 1.15ab	2.55 ± 0.21a
JJ	17.33 ± 1.158a	2.64 ± 0.14a	10.00 ± 1.00cd	1.92 ± 0.19b	10.33 ± 0.58ef	2.09 ± 0.25a
GPG	15.00 ± 0.00ab	2.47 ± 0.17ab	15.33 ± 0.58b	2.43 ± 0.31ab	17.33 ± 0.58a	2.60 ± 0.18a
PLJ	13.00 ± 1.00bc	2.22 ± 0.11ab	8.33 ± 1.53d	1.92 ± 0.07ab	9.67 ± 0.58f	1.94 ± 0.29a
XBH	15.00 ± 1.00ab	2.36 ± 0.09ab	15.33 ± 1.15b	2.50 ± 0.40ab	14.67 ± 0.58bc	2.56 ± 0.15a
CF	11.33 ± 1.15cd	2.34 ± 0.19ab	10.33 ± 1.15cd	2.26 ± 0.28ab	13.33 ± 1.15bcd	2.39 ± 0.25a
DG	11.00 ± 1.00cd	2.32 ± 0.33ab	13.67 ± 0.58bc	2.37 ± 0.16ab	12.00 ± 1.00cde	2.43 ± 0.32a
XYL	9.67 ± 1.52d	2.08 ± 0.06b	8.00 ± 0.00cd	1.91 ± 0.07ab	8.67 ± 0.58f	1.89 ± 0.19a

Stream names: XBX = Xinbaoxiang, H = Haihe, XB = Xiaba, YA = Yaoan, JJ = Jinjia, GPG = Guangpugou, PLJ = Panlongjiang, XBH = Xibahe, CF = Chuangfang, DG = Daguang, and XYL = Xinyunliang. The different letters indicate significant differences (*p* < 0.05)

**Table 4:** Eigen values, *F* values, and *p* values obtained from the partial RDAs testing the influence of the significant parameters on the denitrifying bacterial community composition^*∗*^.

Samples	Environmental variables	Eigen value	% variation explains solely	*F* value	*p* value
*nirK*	PO_4_^3−^	0.21	21	2.37	0.002
	TN	0.19	19	1.32	0.190
	pH	0.10	10	1.12	0.398
	All the above together	0.80			

*nirS*	pH	0.10	10	1.20	0.298
	Temperature	0.11	11	1.20	0.360
	DO	0.08	8	1.10	0.388
	All the above together	0.77			

*nosZ*	Temperature	0.17	17	1.83	0.048
	NO_3_^−^	0.13	13	1.17	0.060
	TP	0.08	8	1.31	0.278
	All the above together	0.83			

^*∗*^Only keeping the first three significant parameters in models of RDAs based on Monte Carlo permutation (*n* = 499). Sum of all Eigen values for both partial RDAs was 1.000.

**Table 5:** Correlations between abundance and parameters for denitrifying genes in the streams.

	*nirK*	*nirS*	*nosZ*	*nirK*/*nirS*	*nosZ*/(*nirK *+ *nirS*)
*nirK*	1				
*NirS*	0.933^*∗∗∗*^	1			
*nosZ*	0.886^*∗∗∗*^	0.770^*∗∗∗*^	1		
*nirK*/*nirS*	−0.011	−0. 225	−0. 1743	1	
*nosZ*/(*nirK *+ *nirS*)	0.349^*∗*^	0.264	0.632^*∗∗∗*^	−0. 396	1
DO	−0.513^*∗∗∗*^	−0.411^*∗*^	−0.420^*∗*^	−0.175	−0.333
pH	0.111	0.201	0.244	0.019	0.021
Temp (°C)	−0.507^*∗∗∗*^	−0.543^*∗∗*^	−0.459^*∗∗*^	0.357^*∗*^	−0.378^*∗*^
ORP	−0.582^*∗∗∗*^	−0.649^*∗∗∗*^	−0.525^*∗∗*^	0.272	0.337^*∗*^
NO_3_^−^ (mg L^−1^)	−0.394^*∗*^	−0.519^*∗∗*^	−0.449^*∗∗∗*^	0.665^*∗∗∗*^	−0.047
TN (mg L^−1^)	0.709^*∗∗∗*^	0.698^*∗∗∗*^	0.676^*∗∗∗*^	0.008	0.426^*∗∗*^
PO_4_^3−^ (mg L^−1^)	0.868^*∗∗∗*^	0.872^*∗∗∗*^	0.875^*∗∗∗*^	0.007	−0.334^*∗*^
TP (mg L^−1^)	0.560^*∗∗∗*^	0.601^*∗∗∗*^	0.601^*∗∗∗*^	0.283	0.377^*∗*^

*∗* is significant at the 0.05 level (two-tailed); *∗∗* is significant at the 0.01 level (two-tailed); *∗∗∗* is significant at the 0.001 level (two-tailed).
